# Synergy of melanin and vitamin-D may play a fundamental role in preventing SARS-CoV-2 infections and halt COVID-19 by inactivating furin protease

**DOI:** 10.1186/s41231-020-00073-y

**Published:** 2020-11-05

**Authors:** Kishalay Paria, Debarati Paul, Trinath Chowdhury, Smritikana Pyne, Ranadhir Chakraborty, Santi M. Mandal

**Affiliations:** 1 Department of Zoology, Vidyasagar University, Midnapore, West Bengal India; 2 Amity Institute of Biotechnology, Amity University, Noida, Sector 125 201313 India; 3 Central Research Facility, Department of Agricultural and Food Engineering, Indian Institute of Technology Kharagpur, Kharagpur, 721302 India; 4 Department of Biotechnology, University of North Bengal, Raja Rammohanpur, Darjeeling, West Bengal 734013 India

**Keywords:** Vitamin-D, Furin, Melanin, SARS-CoV-2, COVID-19, Microbial melanin synthesis

## Abstract

Since the birth of Christ, in these 2019 years, the man on earth has never experienced a survival challenge from any acellular protist compared to SARS-CoV-2. No specific drugs yet been approved. The host immunity is the only alternative to prevent and or reduce the infection and mortality rate as well. Here, a novel mechanism of melanin mediated host immunity is proposed having potent biotechnological prospects in health care management of COVID-19. Vitamin D is known to enhance the rate of melanin synthesis; and this may concurrently regulate the expression of furin expression. In silico analyses have revealed that the intermediates of melanin are capable of binding strongly with the active site of furin protease. On the other hand, furin expression is negatively regulated via 1-α-hydroxylase (CYP27B1), that belongs to vitamin-D pathway and controls cellular calcium levels. Here, we have envisaged the availability of biological melanin and elucidated the bio-medical potential. Thus, we propose a possible synergistic application of melanin and the enzyme CYP27B1 (regulates vitamin D biosynthesis) as a novel strategy to prevent viral entry through the inactivation of furin protease and aid in boosting our immunity at the cellular and humoral levels.

## Introduction

SARS*-*CoV-2, is the root cause for the late pandemic, novel coronavirus disease 2019 (COVID-19). A sick individual experiences mild to serious respiratory problems. Among infected populations, barely any symptomatic patients recoup without hospitalization and the vast majority of them are hospitalized for extraordinary treatment. This is a remarkable worldwide war, where hospitals are in the front line and doctors being commanding officers along with the medical support team, are constantly battling against COVID-19 [1, 2]. By and large, COVID-19 manifests symptoms like other SARS-CoV- infected diseases, advances rapidly towards developing ARDS (acute respiratory distress syndrome) with septic stun, in worst circumstances failure of multiple organs take place because of viral-infection-instigated cytokine storm in the body [3]. The novel corona virus or SARS-CoV-2 is commonly spread via little miniscule droplets liberated into the surrounding environment when the infected persons unguardedly sneeze, cough or even talk with people in close contact [4].

There is no affirmed anti-COVID-19 medication in the existing shelf [5]. A few clinical trials are currently in progress and a few drugs, for example, chloroquine, remdesivir, arbidol, and favipiravir have been tried yet none of them is fruitful altogether to improve the survivability rate [6]. As of now, there are no particular guidelines and treatment regime for COVID-19. Most treatment methodologies are symptomatic and based on supportive therapy. Scarcely any medications have demonstrated great adequacy at the cell level which need further trial and approval. A few antimicrobials including antiviral drugs were utilized to treat COVID-19 patients, for example, blend of remdesivir or lopinavir or ritonavir and chloroquine [7–9] also several drugs are in pipeline [10]. Following application of drugs hostile to viral and other microbes, the shattered natural parity of the gut microbiome further contributes to the progression of morbidity in patients. Corona patients predominantly experience the ill effects of decreasing white-blood corpuscles (WBC) and lymphocytes when there is an urgency of maintaining a threshold level of cytokine level including IL-6 and IL-10 [11]. In this situation, coordinated host-immune based treatments remain decisive to get by against COVID-19.

Nutrient treatment, especially Vit-C and Vitamin D, is a long known practice against the coronavirus affected patients [12]. As of late, phase-3 trials of Vitamin D treatment with different dose management for COVID patients are in progress [13]. Vitamin-D might be a possibly intriguing steady treatment against SARS-CoV-2 infection. Anyway, no logical proof or scientific evidence has been perceived up to this point. Here, a cross-talk between Vitamin D and melanin synthesis pathway has been reported with a fascinating observation where by-products of melanin synthesis unequivocally tie to the dynamic site of human protease furin which is vital for the SARS-CoV-2- mediated disease progression [14].

### Vitamin D: contribution to human health

**Fig. 1:**
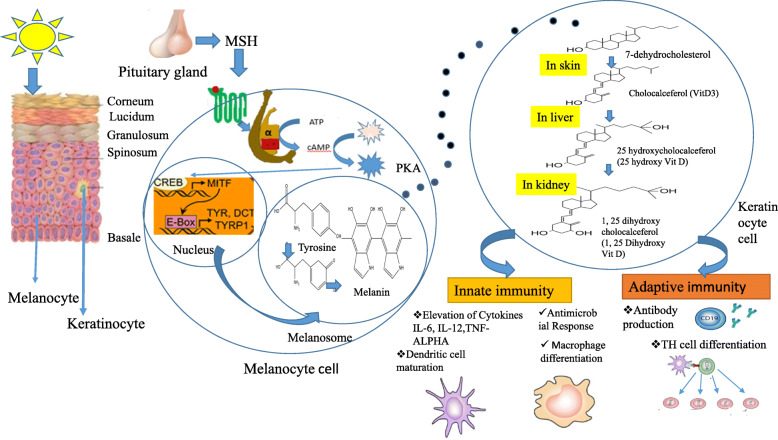
Schematic representation of the cross road between sunlight, vitamin D synthesis, activation of melanocytes and their regulation of immune molecules

**Fig. 2:**
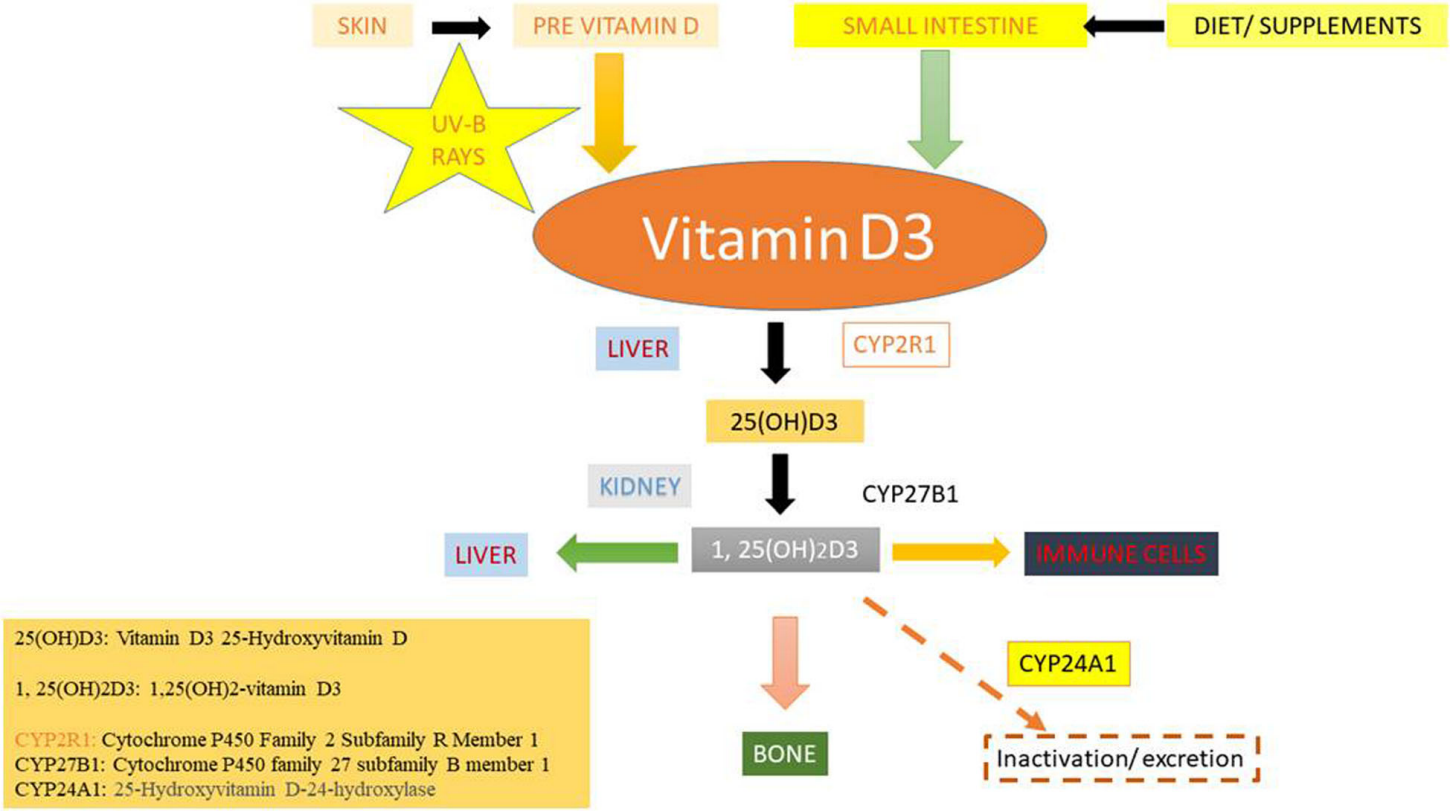
Consequences of Vitmain D in human health and their metabolism in liver. The hydroxylated form, 25 OH vitamin D3 (25 D) in synthesized in liver and converted to most active form, 1,25,dihydroxyvitamin D (1, 25 D) by 1-α-hydroxylase (CYP27B1). CYP27B1, is the key factor to vitamin D biosynthesis

Vitamin D is commonly obtained from food sources or synthesized within human skin [15]. It is widely documented that this vitamin modulates both the adaptive or innate immuity (Fig. 1). Vitamin- D receptors (VDRs) that are displayed on B-cells/ T-cells or on the APCs (antigen presenting cells) can synthesize an active metabolite from vitamin-D. An essential activity of Vitamin D is the maintainance of calcium homeostasis and skeletal health. In the liver, hydroxylated Vitamin D enters a dynamic form, i.e. 25 OH vitamin D3 (also called 25D). Inside the kidney, 25D gets transformed to another dynamic form i.e. -1,25,dihydroxy vitamin D (1, 25 D), also called calcidiol via the action of an enzyme, 1-α-hydroxylase (**CYP27B1**). Subsequently, 24-hydroxylase (**CYP24**) converts 1,25 D to an inactive compound i.e. 1,24,25 vitamin D (Fig. 2). 1, 25 D in active form impacts the intestine and bones after binding to the VDR [16].

A few cross-sectional investigations uncovered that Vitamin D content in the body are directly connected to the progression of diseases such as flu [17], similar to other infections caused by virus for example, HIV etc. [18, 19]. There is an immediate relationship between low levels of vitamin D with diminished resistance and higher rates of viral diseases (Table 1). It is well documented that macrophages use toll like receptors (TLR) to detect lipopolysaccharide (LPS), LPS being a preffered target for bacterial infection. TLR on the macrophages prompts enhancement in expression of both, 1-α-hydroxylase and the VDR. People are increasingly inclined to viral infections of the upper part of respiratory tract, owing to lower vitamin D content, as compared to people who have adequate levels. The level of vitamin D fluctuates with the age, season, sex, race and body mass which is like wise connected with viral infections [32]. The expression of VDR is seen in brain, breast, bone marrow, colon, malignant cells and immunogenic cells other than skeletal and intestine, propose ingthat vitamin D have major protective role by maintaing the homeostasis of calcium in bone tissue as well as increasing the immunity to battle against human pathogens [33].

**Table 1:** List of viral pathogens evidenced for anti-viral feat by the consequence of Vitamin D

Sl no.	Target virus	References
**1**	Hepatitis C virus (HCV).	Gal-Tanamy et al., 2011 [20]
**2**	Respiratory syncytial virus (RSV)	Hansdottir et al., 2010 [21]
**3**	Chronic hepatitis C virus (HCV).	Falleti et al., 2012 [22]
**4**	Rhinovirus replication	Telcian et al., 2017 [23]
**5**	HIV-1	Aguilar-Jimenez et al., 2016 [24]
**6**	Dengue virus replication	Puerta-Guardo et al., 2012 [25]
**7**	Herpes simplex virus-1 (HSV-1) infection	Kumar et al., 2018 [26]
**8**	Epstein-Barr virus	Disanto et al., 2011 [27]
**9**	Rubella virus	Ovsyannikova et al., 2010 [28]
**10**	Hepatitis B virus replication	Farnik et al., 2013 [29]
**11**	Influenza A	Urashima et al., 2010 [30]
**12**	SARS-CoV-2 (Covid-19)	Christianto et al., 2020 [31]

### Vitamin D mediated innate immunity

During respiratory infections, the pathogens go into the respiratory tract and begin to colonize in epithelial cells, also immediately the innate immune and inflammatory signalling network start to respond [34]. Firstly, neutrophils enter the parenchyma cells of the lung and colonization begins within an hour of infection. Subsequently within few days of infection, it effects the natural killer cells, monocytes/macrophages and T cells. RIG- like receptor and TLRs recognize viral pathogen and participate in anti-viral defence system by producing cytokines and type I interferon (IFN). The IFN and proinflammatory cytokines hinder viral replication and translation thereby controlling the infection [25]. These immune molecules additionally actuate viral cleavage, repress viral fusion by the activation of cytolytic cells and stimulate humoral factors, for example, acute-phase proteins, collectins, defensins, including complement proteins [35, 36].

In the presence of viral antigens, macrophages and dendritic cells start processing the CD 4^+^ and CD 8^+^ T cells inpresent in the affected lymphatic nodes. After that T cells move into the infected tissue to intervene pro-inflammatory and cytolytic impacts [37]. Then again, T-helper cell promotes B cell proliferation as well as differentiation into plasma and memory cell, they undergo antibody class switching to synthesize both IgA and IgG [35]. B cell delivering antibody forestalls the entry of virus into cells and prompts phagocytosis by the innate immunogenic cells. Subsequently, the innate and adaptive immune responses can assume cooperative role for protection of individuals from respiratory viral infections [37].

Among all vitamins, only vitamin D is synthesized by the exposure of sunlight (280–315 nm) on the skin. During exposure of sunlight to the keratin rich cutaneous membrane, 7-dehydrocholesterol is finally converted to vitamin D3, calcitriol [38]. Majorly systemic vitamin D is skin- based while a small fraction likewise originates from the dietary supplementation [39]. The intermediate, 1,25(OH)2D3 regulates over 1000 genes after interaction with VDR. Epithelial cells express a critical level of VDR with SNP, related to risk posed by of RSV infection [40, 41]. 1,25(OH)2D3 invigorates maturation of NK cells, neutrophils and macrophages inside the respiratory tract, and furthermore a few antimicrobial peptides (AMPs), for example, cathelicidins and defensins [42]. Such AMPs have shown anti-viral (mainly anti-influenza) impact by associating with hCAP18/LL-37 [43]. Then again, the expression of CD14 and TLR are also likewise affected by 1,25(OH)2D intermediate [44]. The increased activity of macrophages decreases the activity of autophagy during the infection [45]. Autophagy is a cytokine induced cellular homeostasis process. Autophagy is coupled to IFN-α /CXCL10 release to stall viral replication, during infection by Influenza-A [46]. Therefore, vitamin D interceded autophagy restraint can control the respiratory viral disease of lungs. The innate immune response by the action vitamin D become more extensive to energize the movement of myeloid dendritic cells to lymph organs to stimulate specific T_H_ cells/ and B cells [47, 48]. Pro- inflammatory cytokine is likewise hindered by Vitamin D. At the time of influenza-A infection, 1,25 (OH)2D can reduce tumour necrosis factor (TNF)-α and interleukins e.g. IL-8, IL-6, IFN-β, and RANTES in epithelial layer of lungs [21]. Especially viral replication rate, level of cytokine is high in more pathogenic viral strain than less pathogenic strain [49]. Respiratory viral infection rate are lower in summer than winter [47]. Vitamin D can control respiratory viral infection by modulation of adaptive immunity through down regulation of cytokine level of T_H_1 and T_H_2 [50] but upregulation of T regulatory cell [51]. Strikingly, it was seen that Infants who are not exposed to sunlight are found to suffer from lower respiratory tract infection and a low level of 25(OH) D was detected in blood sera [52]. Report showed that sunlight-exposed mother give a high measure of vitamin D to a kid than mother without exposure to sunlight. Sunlight exposure as well as dilatory vitamin D is necessary to maintain foetal growth and development of immunity, along these lines sunlight is an important stimulator of vitamin D synthesis [53].

### Cross-talk between vitamin D and melanin biosynthesis

Human skin spontaneously produces vitamin D during exposure to sunlight. This procedure is a photochemical reaction initiated after 7-dehydrocholesterol present in our epidermis absorbs UV-B and therefore the synthesis depends on the few factors such as UV-B dose, temperature, and lipid environment [54]. Melanin pigmentary system affect vitamin D signalling via the linkage between melanogenic machinery in skin and circulating 25(OH) D of Caucasian individuals [55]. Melanin absorbs UV-B (290–320 nm) and participates in the filtration of light which determines the amount of the UV-B radiation to be penetrated in the skin epithelium [56]. It is confirmed that the world’s racial distribution by latitude is regulated by vitamin D production in individuals [57]. When people migrated from lower latitude to higher latitudes; their skin colour faded due to decreased sunlight. Thus, skin pigmentation is a dominant variable for regulating vitamin D3 synthesis in competition to melanin with 7-dehydrocholesterol [58]. In these conditions, a famous hypothesis “vitamin D-folate hypothesis”, portrays the explanation behind an apparent adaption of human skin shading in UV radiation situations. Vitamin D and folate have diverse sensitivity level against UV radiation, strangely when vitamin D is blended utilizing UVR exposure and afterward folate is degraded. The proposed supposition of “vitamin D-folate hypothesis” is that pigmentation of skin keeps up the cell homeostasis of vitamin D [59]. Several alternative theories restrict this hypothesis. Experts in the area of vitamins do accept theories on converse relationship between a cutaneous pigmentary framework and vitamin D creation [60, 61].

In normal daylight, UVB is the dynamic radiation for vitamin D synthesis over UVA. In an investigation, it is reported that fair complexion individuals need an exposure of 20–30 min and a few times in 7 days to create 20,000 IU of vitamin D3, while a brown complexion individual with high melanin level requires 2–10 fold more exposure time for equivalent level of vitamin D3 [62]. A synergistic action of Vitamin D and melanin in the skin, is fundamentally imperative to screen the levels of 25-(OH) D in youngsters, the pregnant lady just as young and old. Both, melanin and vitamin D have protective role against viral infection as well as bacterial or fungal diseases [63]. Several factors mediate melanin and vitamin D syntheses in the skin. However, information is not adequate on the synthesis of various categories of pigment and their direct correlation with vitamin D. At the point when a patient is being hospitalized with limitation of UVB exposure, the individual must have constraint of vitamin D synthesis. As a matter of fact, the regulation of vitamin D synthesis and involvement of melanocytes with their regulatory activities have not been studied in details under diseased condition.

### Melanin

**Fig. 3:**
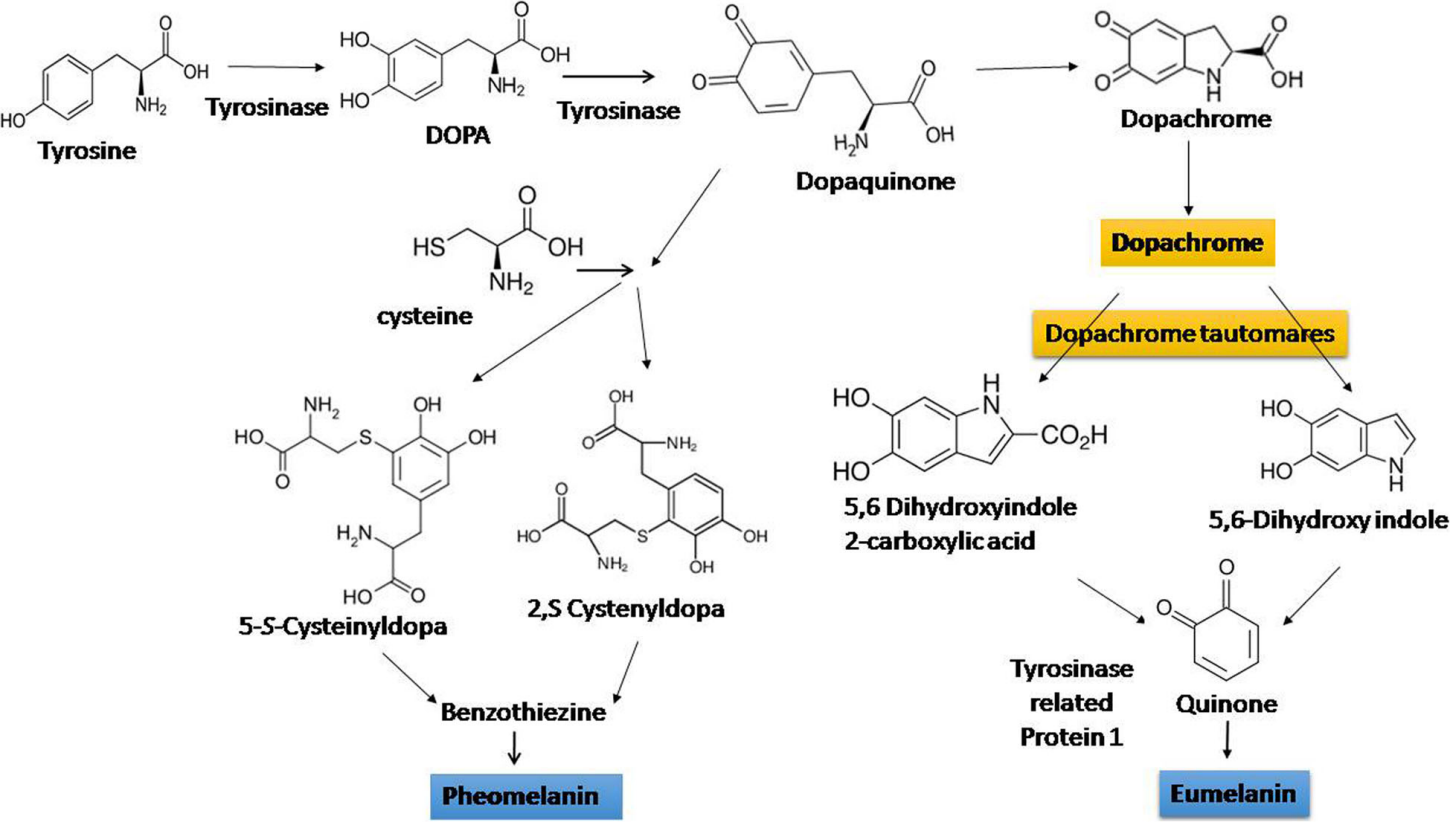
Schematic representation of the melanin synthesis including pheomelanin and eumelanin in human

Indole polymer containing melanin pigments were found in five kingdoms from Monera to Animalia. Melanin goes about as biomarker for evolutionary study. It is considered as most ancient pigments of nature which have been recognizedin fossils of dinosaurs and feathered creatures. From the Jurassic time frame these are found in cephalopod ink sacs [64]. Melanins are three sorts, for example, eumelanins, pheomelanins and allomelanins [65]. Alongside natural conditions, for the most part, two shades are liable for the hue of human skin, for example, eumelanin and pheomelanin. In melanin synthesis pathway, principally the catecholamine precursor 3,4-dihydroxyphenyl alanine (DOPA) is produced from tyrosine, after oxidation which is converted to 3,4-dioxyphenylalanine (dopaquinone) which cyclisize to 5,6-indole quinones and polymerize to melanin (Fig. 3). Dissolvable melanins are orchestrated after L-ß-3,4-dihydroxyphenylalanine (L-DOPA) is oxidized or after L-tyrosine is chemically oxidized [66].

Melanin not just secures photo-induced damage during absorption of high range of the electromagnetic spectrum, but also ensure protection against both chemical and thermal stresses. In this way, melanins are widely utilised for manufacturing photo protective creams/ cosmetics, and also for designing eye glasses or for treating radioactive wastes [67]. Melanin can not only shield our skin from UV radiation-damage and keep up thermoregulation, it also aids in managing stress response, metabolism, and immunity [68]. Prior reports confirm that both, alfa -melanocyte stimulating hormone and melanin-concentrating hormone synthesizing gene of vertebrate are highly conserved, that bind to MRC (melanocortin receptor) of tissue and liable for different physiological activities including defence against parasitic infection [69]. Then again, melanocortin ligand (alfa MSH and ACTH) helps in the expression of MHCI [70].

Along these lines, melanin is every now and again utilized as healthcare material as it have cancer prevention properties, antiviral, antimicrobial, antiinflammatory, antitumor, immuno-stimulating, and radioprotective activities [71]. Synthetic melanin under in-vitro condition was found to inhibit replication of HIV-1or HIV-2 and impeded HIV-1 envelope surface glycoprotein but did not interfere with the activity of reverse transcriptase enzyme [66] and is additionally used for treating metastatic melanoma in human [67]. Therefore, it is accepted that melanin pigment has a significant preventive role against both malignant growth and infectious diseases.

### Furin mediates the pathogenesis of cancer and viral infections

In excess of 500 proteases are accounted for in human genome having significant job as molecular scissors in all physiological process. Proteolytic cleavage regulates several physiological and pathogenesis pathways leading to either health and disease. One of the generally significant and ubiquitously expressed proteases is the serine protease furin [72]. Furin is responsible for the activation of several virus particles. Viruses of enveloped proteins, just as non-enveloped, are processed by this protease along with other proteases for the entry for producing mature virions that are ready for infection. Hormones, growth factors, cytokines, and receptors are the mammalian substrates of furin and unusual activity of furin is directly associated with a multiplicity of dieases, including cancer and viral or bacterial infections [73].

Furin has a place with the group of exceptionally explicit, calcium-subordinate proprotein/prohormone convertases (PCs) [74]. This endoproteinases highlighted a synergist area of homology to subtilisin and initiate an enormous number of emitted proteins by constrained proteolysis. Furin is a sort I transmembrane serine-protease that is universally communicated and cycles from trans-Golgi systems to cells, via the endosomal framework. In warm-blooded animals, the PC family grasps seven individuals that divide after different fundamental deposits at the site of cleavage (R/K)Xn(R/K)↓ (here “↓” denotes scissile peptide security), with furin especially perceiving the sequence R-X-K/R-R↓ [75]. This exceptional succession explicit cleavage is basic for the initiation of various PC substrates.

Although, furin is normally expressed by various cells, its mRNA and protein levels fluctuate depending on the type of cells/ tissues; significant quantities being present in bone marrow, salivary glands, and liver while, in muscle cells furin production is comparatively lower [76]. The pro-peptide of furin is transferred to the *trans*-Golgi network (TGN) from endoplasmic reticulum, during which autoproteolytic process occurs in two steps such that furin becomes enzymatically active [77]. Simultaneously, N-linked oligosaccharides are incorporated and the peptide is trimmed. Since furin levels are elevated in the TGN, it can be transferred to the surface of cells and back to the TGN, via the endosomal pathway [78]. At last, furin is shed and discharged as extracellular protein after the proteolytic cleavage of its catalytic membrane-bound domains [79]. The capability to act upon a range of cellumar substrates within the cell or in the extracellular spaces is related to the ubiquitous presence of furin, not only within the TGN and endosomal- compartments, but also on cellular surfaces.

Mostly glycoproteins present on the viral envelope are proteolytically severed before entering host cells. May a times, viruses utilise cellular enzymes e.g. trypsin or subtilisin-like endo-proteases for such actions. Furin which is a subtilisin-like protease recognise and cleave at polybasic locii; a trypsin-like protease however, perceives mono-basic sites to cut next to any single Arg or Lys residue [80]. Various reports have shown that glycoproteins belonging to the coat of several viruses (Borna, Pneumo, Orthomyxo, Herpes, Flavi, Toga, Bunya, Filo, Paramyxo, Corona, and Retroviridae) are cleaved by Furin, although these viruese are evolutionarily divergent.

### In silico analysis among furin and melanin intermediates

Albeit viral furin substrates by and large contain polybasic canonical cleavage site, its active site binding pocket is conserved in many species. Viral glycoproteins and furin protein, both enter the secretory pathway, allowing proteolytic cleavage at various times during replication of viral genome. The proteins coat of few viral strains are produced separately and not along with the genome in producer cells, while in others the protein envelop is extracellularly processed before the virus attacks another target host cell. A good number of viruses utilise furin and other proprotein convertases (PCs) in order to regulate their entry into host cell and develop high pathogenicity [72]. It is well documented that several growth factors, receptors, matrix metalloproteinases and viral envelope glycoproteins are involved in the conversion to their bioactive forms [81, 82]. Recently, in silico to in vitro strategies are undertaken to hinder the furin activity for SARS-CoV-2 spike glycoprotein cleavage repression [83]. Therefore, furin is a taget molecules to halt the entry a number of viruses. There is a direct correlation was also observed between furin and melanosome biogenesis. It was evidenced that the intralumenal fibrils are required to cleave the Pmel17 by a furin-like proprotein convertase (PC). The cleavage of Pmel17 liberates a lumenal domain fragment that helps to regulate the melanosome biogenesis by controlling the fibrillogenic activity [84].

The catalytic domain of furin binds to the target site of catalytic triad (ASP153, HIS194, SER368) with a distinguished oxyanion hole (ASN295). Apart from that, the residue from SER253 to PRO256 likewise demonstrates a strong affinity to small molecule to bind to furin. The accompanying sections feature the interaction of furin with few important intermediates (available structures in PubChem) of melanin biosynthesis pathway.

### Energy minimization and molecular docking

Protein Data Bank file for Human Furin (PDB ID: 4RYD) was utilizedas receptor molecule and Melanine (PubChem CID:6325610), Eumelanine (PubChemCID:102582077), L-DOPA (l^− 3^,4-dihydroxyphenylalanine) (PubChem CID:6047), L-Dopaquinone (PubChemCID:44229226),was taken as ligand molecule for docking. Each molecule was subjected to energy minimization using ChemBio3DUltra 13.0 software, a high quality workstation where MM2 energy minimization of each molecule was identified with stable molecular conformation. Least RMS gradient taken was 0.010. Studies on docking of Melanin, Eumelanine, L-DOPA and L-Dopaquinone with Human Furin was performed using iGEMDOCK v2.1 software by using a basic algorithm to perform automated dockings. The software called AutoDockVina was additionally utilized analysis of results obtained after molecular docking. This software used Pyrex tools or Auto-Dock Tools (ADT) [85]. Gasteiger charges were determined after removing water residues from macromolecules. The ligands and macromolecules were fed into the Pyrex tool [86]. Finally, ligand and receptor files were exported as “pdbqt” format files.

### Molecular docking analysis and self-protective benefit

**Fig. 4:**
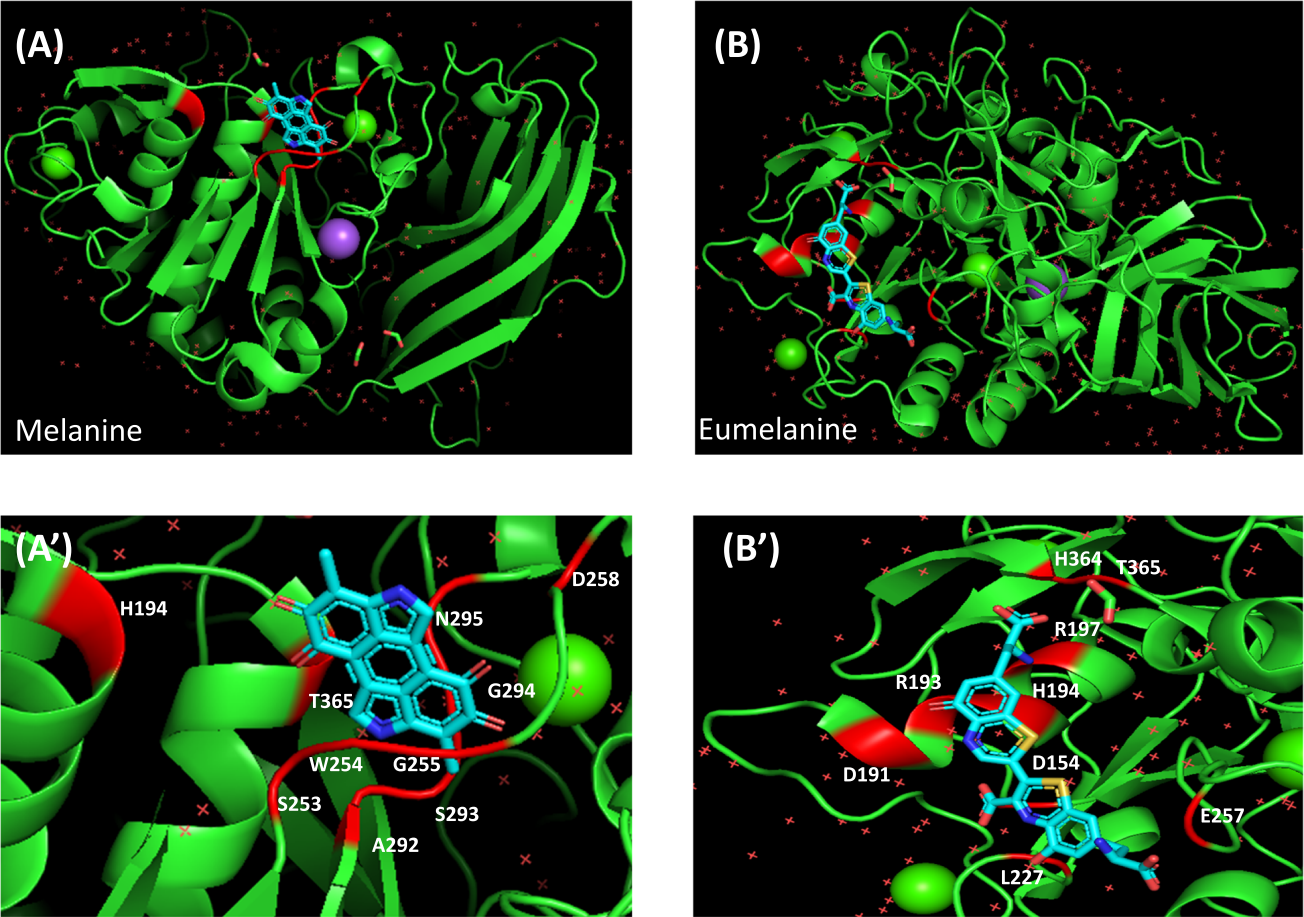
Docked image ofHuman Furin (PDB ID: 4RYD) with Melanine viewed in in PyMO (**A**). Zoomed image of ligand binding site of Furin-Melanine complex (**A’**).Docked image of Human Furin (PDB ID: 4RYD) with Eumelanine viewed in in PyMO (**B**). Zoomed image of ligand binding site of Furin-Eumelanine complex (**B’**)

**Fig. 5:**
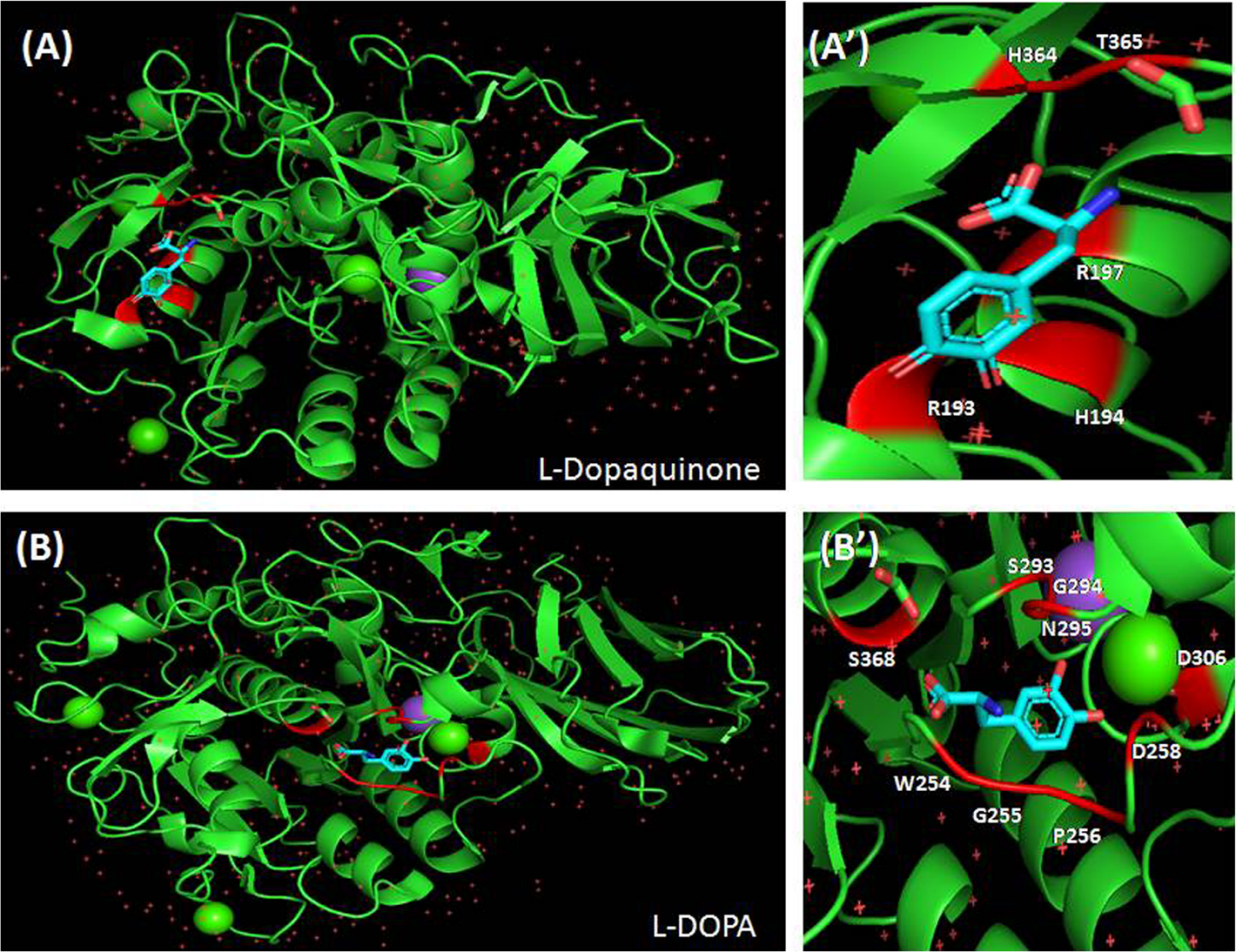
Docked image ofHuman Furin (PDB ID: 4RYD) with L-Dopaquinone viewed in in PyMO (**A**). Zoomed image of ligand binding site of Furin-L-Dopaquinone complex (**A’**).Docked image of human Furin (PDB ID: 4RYD) with L-DOPA viewed in in PyMO (**B**). Zoomed image of ligand binding site of Furin-L-DOPA complex (**B’**)

From the results of molecular docking, it was observed that melanin, eumelanine, L-dopaquinone and L-DOPA emphatically bind with the active site of furin protein and therefore, forestalling the viral entry unwaveringly. In silico docking investigation of melanin with furin protein clearly delineates a binding affinity of − 95.25 kcal/mol (Table 2). Melanin interacts with the residues-HIS194, ASP258, ALA292, SER253, TRP254, GLY255, SER293, GLY294, ASN295, THR367 of furin protein. From Fig. 4A and A**’**, the interaction of melanin with the residues SER253, TRP254 and GLY255, the inhibitor binding site, where melanin binds alongside the single residue of catalytic triad (HIS194) with the oxyanion opening (ASN295) of furin protein. Another docking was performed with eumelanine with furin protein which shows strong binding affinity of − 119.51 kcal/mol (Table 2). Eumelanine binds with the residues-ARG197, ASP153, ASP191, ARG193, ARG197, GLU257, HIS364, THR365, HIS194, LEU227 of furin protein (Fig. 4B and B**’**). In this interaction study, the interacting residues ASP153 and HIS194 are part of catalytic triad where eumelanine emphatically binds strongly. In the docking study of L-Dopaquinone with furin protein, the binding free energy was determined to be − 77 kcal/mol (Table 2). L-dopaquinone interact with the six residues, ARG197, ARG193, HIS194, ARG197, HIS364, andTHR365, of furin protein (Fig. 5A and A**’**). Here, additionally one of the residues of catalytic triad (HIS194) was associated with the interaction study. Docking study of L-DOPA with furin protein additionally uncovers a binding affinity of − 77.15 kcal/mol (Table 2). The interaction of L-DOPA with furin obviously shows an involvement of PRO256, ASP258, SER293, GLY294, ASN295, ASP306, SER368, TRP254, GLY255 residues of furin (Fig. 5b and b**’**). The residues TRP254, GLY255, PRO256 are the inhibitor binding site of furin protein where L-DOPA binds alongside the association of one residue of catalytic triad (SER368).

**Table 2:** Determination of binding free energy of docking interactions between melanine, eumelanine, l-dopaquinone and l-dopa with human furin (4RYD) using iGEMDOCK

Receptor	Ligand	Binding Free Energy (kcal/mol)	Van der waal Energy (kcal/mol)	Hbond Energy (kcal/mol)	Electrostatic Energy (kcal/mol)	Ligand inding Sites
**4RYD:A – Human Furin**	Melanine	−95.25	− 79.46	−15.79	0	HIS194, ASP258, ALA292, SER253, TRP254, GLY255, SER293, GLY294,, ASN295, THR367
**4RYD:A – Human Furin**	Eumelanine	−119.51	−93.09	−23.45	− 2.98	ARG197, ASP154, ASP191, ARG193, ARG197, GLU257, HIS364, THR365, HIS194, LEU227
**4RYD:A – Human Furin**	L-Dopaquinone	−77	−41.99	−28.26	−6.75	ARG197, ARG193, HIS194, ARG197, HIS364, THR365
**4RYD:A – Human Furin**	L-DOPA	−77.15	−46.9	−29.72	−0.53	PRO256, ASP258, SER293, GLY294, ASN295, ASP306, SER368, TRP254, GLY255

### Availability of melanin and perceived opportunity for pharmaceutical/ biotech-industry

**Fig. 6:**
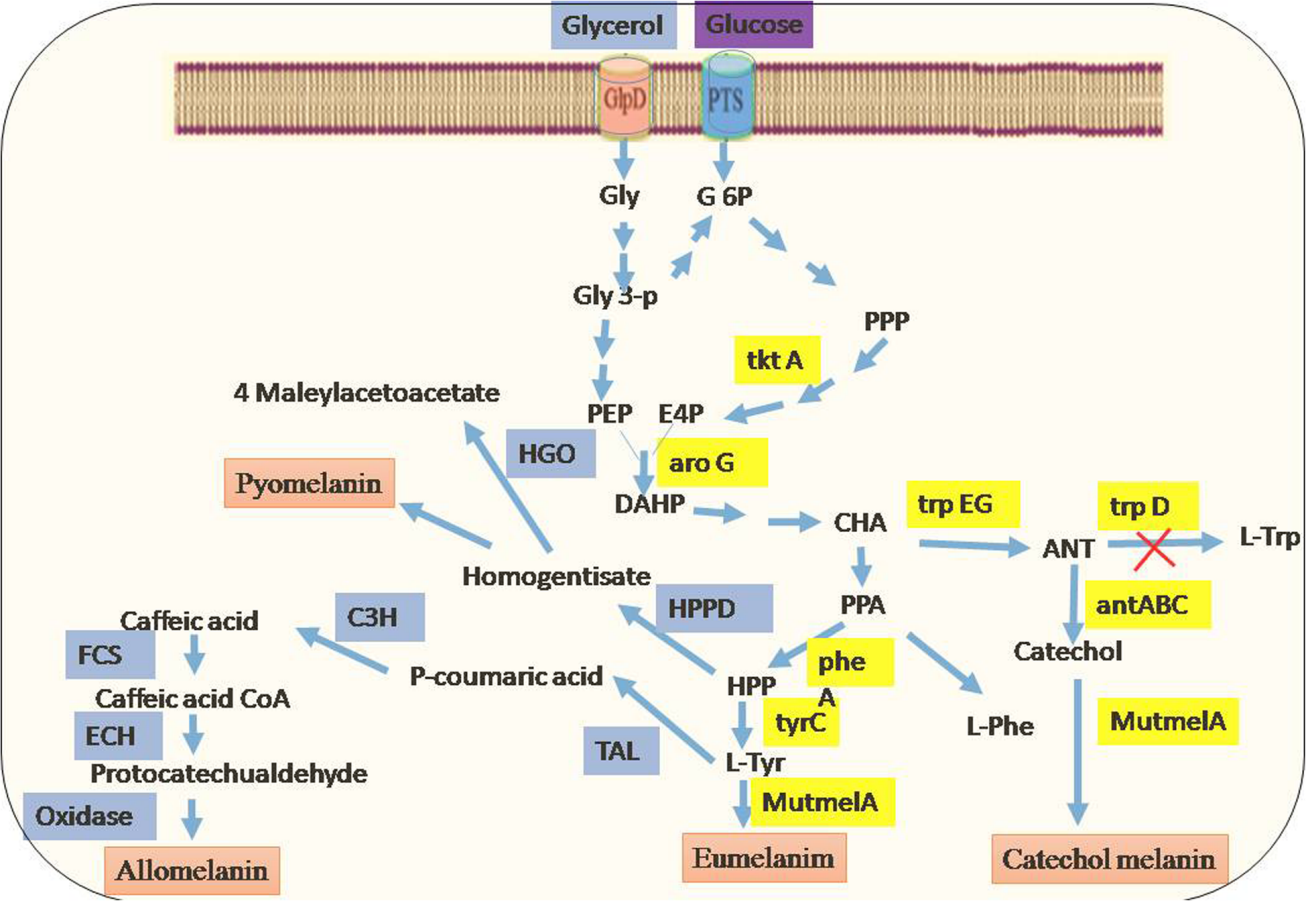
Biosynthesis pathway of melanin in microbes

As the tremendous preventive role of melanin in curbing the spread of several diseases could be perceived (Table 3), a real opportune-market of pharmaceuticals will be ensuing very shortly. Melanin synthesized in skin with the aid of sunlight, is impracticable for a patient to replenish during hospitalization. In case of long run, the genetic level of expression varies among individuals, races and seasonal. Therefore, the availability of melanin is an significant issue about its availability in healthcare application as ‘preventive medicine’. Other than mammals, melanin from microbial sources is available in the market [92]. Next to chemical-synthesis, it can be resourced from many living entities including plant, animal, fungi and bacteria. As of late, eco-friendly microbe-derived melanin has stood out as a biotechnology option in contrast to synthetic/chemical production [93]. By and the large microbial melanin- synthesizing enzyme belongs to laccase and tyrosinase group. The tyrosinases are prevalently associated with melanogenesis. In view of amino acid sequences and catalytic activity, microbial tyrosinases belong to five main classes [94]. Although, tyrosinases (mono-oxygenases) contain dinuclear copper catalytic centre, which catalyse ortho-hydroxylation of mono-phenols (cresolase activity), it can also oxidize catechols (catecholase activity) for synthesis of ortho-quinone. Microbial biosynthetic route of melanin production is summarized in Fig. 6**.** Be that as it may, tyrosinase of a few microorganisms, for example, *Rhizobium etli, Bacillus megaterium,* and *Bacillus thuringiensis* don’t require copper for activation of chaperone protein. Then again, another melanogenic enzyme laccase (copper-dependent oxidoreductases) is principally found in plant, fungi and bacteria [95].

**Table 3:** Definite action of melanin against bacterial and viral pathogens

Anti-bacterial	References	Anti-viral	References
*Escherichia coli, Salmonella typhi,, Vibrio parahaemolyticusListeria monocytogenes, Bacillus megaterium* *Staphylococcus aureus*, *Escherichia coli, Lactobacillus vulgaris, Proteus mirabilus, Vibrio cholerae, Stapylococous aureus, Salmonella paratyphae, Klebsella oxytoca.*	Xu et al., 2017 [87] Vasantha-bharathi et al., 2011 [88]	Influenza Virus Human immunodeficiency virus types 1 and 2 (HIV-1 and HIV-2) Herpes simplex type 2, and vaccinia.	Guobing et al., 1999 [89]; Ashkinazi et al.,2019 [90] Sidibe et al., 1996 [91]; Montefiori & Zhou., 1991 [66]; Ashkinazi et al.,2019 [90] Ashkinazi, et al., 2019 [91]

### Melanin from bacteria

Like mammals, bacteria are also capable of synthesizing melanin [67]. Several bacterial genera, such as *Aeromonas, Bacillus, Azotobacter, Legionella, Proteus, Micrococcus, Mycobacterium, Azospirillum, Pseudomonas, Rhizobium, Shewanella, Streptomyces, Escherichia*, *Bacillus, Klebsiella* and *Vibrio.* have been reported to produce endogenous melanin [64]. *Streptomyces* spp. has melanin operon (*mel*C) constituted of *mel*C1 and *mel*C2 genes that codes for apotyrosinase and tyrosinase enzyme repectively [93]. A group of bacteria is known to produce melanin from L-tyrosine using the key enzyme 4-hydroxyphenylacetic acid hydroxylase [96]. Furthermore, bacterial genes were used in the form of reporter genes to screen recombinant bacterial strains, for synthesis of melanin [97, 98]. A list of selected bacterial and fungal origin of melanin production is provided in Table 4.

**Table 4:** Enhanced melanin production from microbial in origin at a glance

Bacterial strain	References	Fungal strain	References
*Actinoalloteichus* sp.MA32*Streptomyces*sp., *Vibrio cholerae* and *Shewanella col welliana*	Manivasagan et al., 2013.) [99] Vasanthabharathi et al., 2011 [88]	*Cryptococcusneoformans, S. schenckii, P. brasiliensis, Pneumocystis sp, Aspergillus fumigatus, Alternaria alternate, Cladosporium sp. Yarrowiali polytica* *Exophiala* (*Wangiella*) *dermatitidis*, *Cladosporiumcarrioni*, and *Fonsecaea pedrosoi* *Spergillusnidulans*, *Aspergillus niger*, *Exophiala jeanselmei*, *Fonsecaea compacta*, *Hendersonula toruloidii*, *Phaeoannellomyces wernickii*, *Phialophora richardsiae*, *P. verrucosa*, *Xylo hyphabantiana, Histoplasma capsulatum*	Nosanchuk et al., 2006 [100] Apte et al., 2013 [101]. Romero-Martinez et al., 2000 [102] Jacobson, 2000 [103] Gómez and Nosanchuk, 2003 [104]

### Melanin from fungi

Several fungi can produce appreciable amount of melanin from dihydroxynaphthalene (DHN), γ-glutaminyl-4-hydroxybenzene, HGA, tyrosine and catechol. The habitat of about 80% of melanin producing endophytic fungi, in Antractica, is a herbaceous plant, *Deschampsia Antarctica* Desv (Poaceae). Melanized fungi, for example, *Trimmatostroma salinum, Hortaea werneckii, Aureo basidium pullulans*, *Phaeotheca triangularis,* and *Cladosporium* sp. live in salterns [105]. Some melanin producing fungi are found in habitats polluted with heavy metals and unsaturated hydrocarbons from industries and urban wastes [92]. *A. fumigatus* by the virtue of its ability to synthesize dihydroxynaphthalene (DHN) melanin and mobilize the same in the gray-green colour conidia, can inhibit many pathogens. DHN melanin,the product of *pksP* gene, has been shown to protect *A. fumigatus* from reactive oxygen intermediates (ROI). PksP inhibit the lysosome - phagosome fusion to destroy the conidia of *A. fumigatus* [106]. *Cryptococcus neoformans*, *Aspergillus fumigatus* and *Pneumocystis cariniietc* are the major fungal strains reported to produce melanin [107]. Besidesphotoprotection and antioxidant activities, fungal melanins arecapable of rendering resistance against several biotic, abiotic, and radiation stresses, such as antifungal agents, oxidizing agents, salinity, draught, heavy metals, thermal, UV & electromagnetic radiation [106–108]. Human melanin has structural similarity with fungal melanin, more so, melanin extracted from the fungus, *Cryptococcus neoformans*, has been used for the production of monoclonal antibodies (mAb) for treating patients suffering from metastatic melanoma; this monoclonal antibody is capable of binding tohuman melanin [98].

### Recombinant melanin production

Alongside wild type bacteria, many recombinant bacterial strains were constructed for production of melanin. *E. coli* was utilized as a host for producing the first recombinant melanin by cloning and expression of specific genes of actinomycete *Streptomyces antibioticus*. Primarily *mel*, and ORF438*S* genes are involved in melanin production [67]. The modified strain of *E. coli* produced eumelanin at 30 °C in the culture medium following consumption of L-tyrosine [108]. Synthetic amino acids, e.g., L- tyrosine ethyl ester and N-acetyl-L-tyrosine have been propsed for use melanin synthesis by *S. antibioticus tyrosinase* [109]. *E. coli* strain JM109 was used to clone the *mel* gene of *S. antibioticus* and phage T5 promoter and lac operators were used for transcription [67]. In laboratory culture condition, recombinant strain of *Streptomyces kathirae* SC1 was also used to produce considerableamount of melanin by tuning the expression of melC gene [93].

### Cross-talk between vitamin-D and furin protease

The function of our inherint immune system depends on vitamin D content that in turn leads to the protection of our health and well being. As discussed previously macrophages attach to LPS, during bacterial infections, viaTLRs, leading to increase in expression of CYP27B1 (1-α-hydroxylase) and the VDR (Fig. 2). Individuals showing low levels of vitamin D (< 30 ng/ml) were bound to complain about constant infections in the upper respiratory tract than individuals possesing adequate Vitamin D levels, depending on their, age, gender, body mass, race and season [32].

**Fig. 7:**
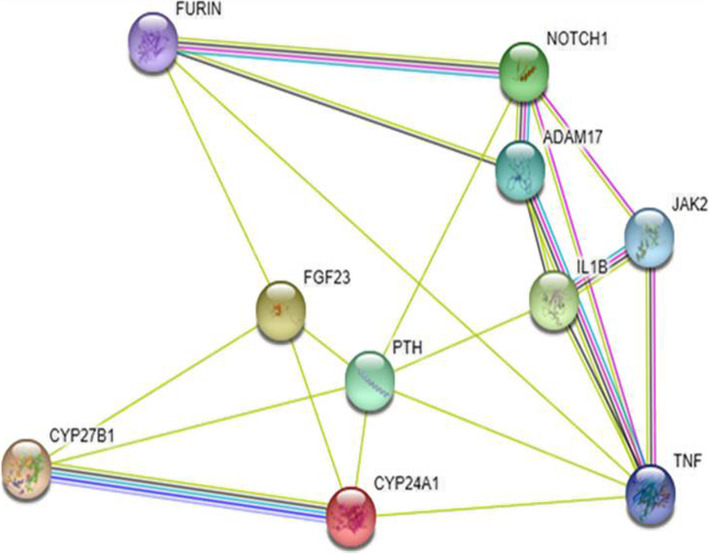
The diagram shows the probable associations between various proteins (analysed using STRING software). The main nodes are formed by FGF23, CYP27B1, CYP24A1, PTH, FURIN, etc. The diagram indicates the importance of vitamin metabolism involving FGF23, CYP27B1, CYP24A1 and the regulation of Furin ultimately, which in turn is responsible for entry of coronavirus into the cell by interaction with TMPRSS2

VDRs are expressed by the brain cells, bone marrow, breast cells and skeletal and intestininal cells. This suggestes that vitamin D may have a role to play, other than the established function of maintaining calcium and bone homeostasis [110]. Furin (ubiquitous endoprotease present within constitutive secretory pathways) activity in other pathways (parathyroid hormone processing, pro-factor IX, etc) is controlled negatively by both, calcium levels and vitamin D in the secretory pathway [111–113]. Increased Furin activity enhances the role of TMPRSS 2 in viral-entry intothe host cells. Negative regulation by Vitamin D may bring down the furin activity and associated TMPRSS2 action thereby decreasing the chances of coronavirus infection (Fig. 7).

## Conclusion and future perspective

Melanin is an useful polymer classified as a natural product having numerous potential applications in the industry based on bioresources including plants, microbes, animal cells and from semisynthetic blends. Skin pigmentation is, in fact, a predominant variable regulating the production of vitamin D3 under conditions of low degrees of solar-illumination since melanin retains UV photons in rivalry with 7-dehydrocholesterol. A few examinations have indicated that lower amounts of vitamin D is correlates with not only an increased susceptibility to acute infections, but also with chronic infections (such as HIV infection) in some cases. Taking vitamin D supplements may improve ones response to treatment for diseases caused by virus or bacteria, such as chronic hepatitis C or pulmonar tuberculosis. No benefit could be derived from solo treatment using vitamin D in reducing pulmonary infections. The dose of vitamin D supplemention along with antiviral or antibacterial drug is not well defined. Unmistakably, 1,25(OH)2D has numerous immunomodulating properties which might decrease risks of respiratory infections caused by viruses. As discussed in this review, one toward this path can be founded on the applications of natural melanin bioextracted from novel melanogenic organisms. We have reviewed the applicability of vitamin D and D3 for modulating immunity in human beings, which is suggests using vitamin D/D3 to treat Covid-19. We have inferred three main issues- First, vitamin D induces immunity. Second, vitamin D production may be influenced by melanin. Third, both vitamin D and melanin may have significant impact in management of COVID-19. Determining the right category of melanin pigment (eumelanine or pheomelanin) to be used in specified amounts for vitamin D production, its mobility in the body, and initial hydroxylation, should be studied in future. We trust that this short review article will be useful in shaping the future course of treating COVID-19.

## Abbreviations

AMPs: Antimicrobial peptides
ARDS: Acute respiratory distress syndrome
COVID-19: Novel coronavirus disease 2019
IFN: Type I interferon
DOPA: 3,4-Dihydroxyphenyl alanine
L-DOPA: L-ß-3,4-dihydroxyphenylalanine
SARS-CoV-2: Severe acute respiratory syndrome coronavirus 2
CYP27B1: 1-α-hydroxylase
Vitamin C: Vit-C
Vitamin D: Vit-D
VDR: Vitamin- D receptor
CYP24: 24-Hydroxylase
TLR: Toll-like receptors
LPS: Lipopolysaccharide
TNF: Tumour necrosis factor
UV-B: Ultra violet-B
MRC: Melanocortin receptor
MSH: Melanocyte-stimulating hormone
ACTH: Adrenocorticotropic hormone
MHC1: Major histocompatibility complex (MHC) class 1
Pcs: Prohormone convertases
TGN: Trans-golgi network
TMPRSS2: Transmembrane protease serine 2 precursor
